# Dysbiosis of Gut Microbiota Is Associated With the Progression of Radiation-Induced Intestinal Injury and Is Alleviated by Oral Compound Probiotics in Mouse Model

**DOI:** 10.3389/fcimb.2021.717636

**Published:** 2021-10-25

**Authors:** Tian-Shu Zhao, Li-Wei Xie, Shang Cai, Jia-Yu Xu, Hao Zhou, Lin-Feng Tang, Chao Yang, Shuguang Fang, Ming Li, Ye Tian

**Affiliations:** ^1^ Department of Radiotherapy and Oncology, The Second Affiliated Hospital of Soochow University, Suzhou, China; ^2^ Institute of Radiotherapy and Oncology, Soochow University, Suzhou, China; ^3^ State Key Laboratory of Radiation Medicine and Protection, School of Radiation Medicine and Protection, Medical College of Soochow University; Collaborative Innovation Center of Radiation Medicine of Jiangsu Higher Education Institutions, Soochow University, Suzhou, China; ^4^ Department of Nucleus Radiation-Related Injury Treatment, PLA Rocket Force Characteristic Medical Center, Beijing, China; ^5^ Wecare Probiotics (Suzhou) Co., Ltd, Suzhou, China

**Keywords:** ionizing radiation, intestinal injury, dysbiosis, biomarker, probiotics

## Abstract

The acute radiation-induced intestinal injury (RIII) has raised much concerns and is influenced by non-cytocidal radiation effects including the perturbations in gut microbiota. Although a number of studies have reported alteration in gut microbiota following radiation, little is known about its dynamic variation in the progression of acute RIII. In this study, mouse model were treated with total body irradiation (TBI) of 0, 4, 8 and 12 Gy, and the intestinal tissues and fecal samples were collected at 6 h, 3.5 d and 7 d post radiation. We found that the intestinal injuries were manifested in a radiation dose-dependent manner. Results from *16S rRNA* gene sequencing demonstrated that the diversity of gut microbiota was not significantly affected at the prodromal stage of acute RIII, after 6 h of radiation. At the critical stage of acute RIII, after 3.5 d of radiation, the composition of gut microbiota was correlated with the radiation dose. The Pearson’s correlation analysis showed that the relative abundances of phylum Proteobacteria, genera *Escherichia-Shigella* and *Eubacterium xylanophilum_group*, and species *Lactobacillus murinus* exhibited linear correlations with radiation dose. At the recovery stage of acute RIII, after 7 d of radiation, the diversity of gut microbiota decreased as a whole, among which the relative abundance of phyla Proteobacteria and Bacteroides increased, while that of phylum Tenericutes and genus *Roseburia* decreased. The intra-gastric administration of compound probiotics for 14 days improved the survival duration of mice exposed to 9 Gy TBI, alleviated the intestinal epithelial injury and partially restored the diversity of gut microbiota. Our findings suggest that acute RIII is accompanied by the dysbiosis of gut microbiota, including its decreased diversity, reduced abundance of beneficial bacteria and increased abundance of pathogens. The gut microbiota cannot be used as sensitive biomarkers at the prodromal stage in acute RIII, but are potential biomarkers at the critical stage of acute RIII. The dysbiosis is persistent until the recovery stage of acute RIII, and interventions are needed to restore it. The administration of probiotics is an effective strategy to protect against acute RIII and subsequent dysbiosis.

## Introduction

Due to the accidental exposure caused by the usage of radioactive or nuclear devices in nuclear power plants and terrorist attacks, the human beings may be exposed to high dose ionizing radiation (IR) in a short time, termed as acute radiation syndrome (ARS), and acute gastrointestinal syndrome is the main cause of early death in ARS (Kamiya et al., 2015). Besides, 60-90% of the patients develop the symptoms of acute intestinal toxicity due to the radiotherapy for abdominal and pelvic malignant tumors, significantly affecting the quality of their lives and interrupting their treatment’s plan (Kumagai et al., 2018). Unfortunately, there is no current objective and effective strategies for the diagnosis and treatment of acute radiation-induced intestinal injury (RIII).

The patho-biological changes, which occur during RIII, are complicated, involving multiple tissues and cell types in the gut (Hauer-Jensen et al., 2014). Based on the development of *16S rRNA* gene sequencing technology for decades, a number of studies have reported alteration in gut microbiota following radiation (Goudarzi et al., 2016; Cui et al., 2017; Reis Ferreira et al., 2019; Wang Z. et al., 2019), thereby influencing intestinal response to radiation (Gerassy-Vainberg et al., 2018; Kumagai et al., 2018; Wang Z. et al., 2019). The perturbations in the composition and functions of gut microbiota, which are detrimental to host, are called dysbiosis (Levy et al., 2017; Sommer et al., 2017). Although the composition of gut microbiota is affected by multiple factors, such as geographical location, dietary, age and rhythm (Cresci and Bawden, 2015), several clinical studies conducted in different regions have reported dysbiosis in the gut microbiota following abdominal and pelvic radiotherapy. A study by Nam et al. found that the overall composition of gut microbiota was altered after pelvic radiotherapy; in particular, the phylum Firmicutes decreased by 10% and phylum Fusobacteria increased by 3% after radiotherapy (Nam et al., 2013). Wang et al. found that the pelvic radiotherapy caused a reduction in the alpha diversity of gut microbiota and an increase in the relative abundance ratio of Firmicutes to Bacteroidetes (Wang et al., 2015). Wang et al. studied the alpha diversity of gut microbiota and found a decrease in the relative abundance of Bacteroides and an increase in that of Proteobacteria among patients with radiation enteritis (Wang Z. et al., 2019). Laboratory studies also found that the IR significant altered the gut microbiota in mice. Gerassy-Vainberg et al. found an increase in the relative abundance of Bacteroidetes and Proteobacteria, and a decrease in that of Firmicutes, where the alteration in the composition of gut microbiota was more significant at 6 weeks post radiation as compared to 2 weeks post radiation in radiation-induced proctitis mouse model (Gerassy-Vainberg et al., 2018). Goudarzi et al. showed that the fecal microbial diversity was lower on day 3 after 5 Gy total body irradiation (TBI) and restored to normal on day 30 in mice (Goudarzi et al., 2016). Li et al. and Lu et al. reported that the composition of gut microbiota was substantially altered at 3.5 d after 9 Gy TBI and at 5 d after 15 Gy whole abdominal irradiation, respectively, resulting a decrease in the relative abundance of Bacteroidetes and an increase in that of Proteobacteria, while the diversity of gut microbiota was not affected in alpha diversity analysis (Li et al., 2019; Lu et al., 2019).

The above studies show disparities in the alteration of gut microbiota following radiation. Apart from the factors such as radiation dose and field, the different taxonomic classifications and nomenclature of bacteria and circadian rhythmicity, affecting the composition of gut microbiota (Thaiss et al., 2014; Liu et al., 2021), might be crucial determinants, leading to apparent inconsistency. The rhythmic oscillations in operational taxonomic units account for about 60% of the microbial composition and result in time-of-day-specific taxonomic configurations (Thaiss et al., 2014). The relative abundances of Bacteroidetes and Firmicutes, which are the two most abundant phyla in mammalian gut microbiota, varied in the 24 hours of a day (Liang et al., 2015). For instance, Bacteroidetes were the most abundant at 11:00 PM and the least abundant at 7:00 AM (Liang et al., 2015). Oscillations in the relative abundance of the multiple genera of Firmicutes, including *Clostridiales* spp., *Turicibacter*, *Ruminococcaceae* spp., *Clostridia* spp., and *Lachnospiraceae* spp. etc., were observed (Liang et al., 2015). However, in the above-mentioned studies, the timing for fecal sample collection was not specific. From this aspect, the comparison of findings from previous studies seems unreasonable to some degree. Moreover, the above studies did not systematically explore the changes in gut microbiota at the different stages of disease and different degrees of injury in RIII.

Herein, in order to discern a clear signature for the association of gut microbiota with acute RIII, the fecal samples of C57BL/6 mice were taken at 6 h (prodromal stage), 3.5 d (critical stage) and 7 d (recovery stage) post 0, 4, 8 and 12 Gy radiation in correspondence with the different severity of ARS (Broin et al., 2015) and analyzed using *16S rRNA* gene sequencing technology. This study suggested that the fecal bacteria could not be used as sensitive biomarkers at the prodromal stage of acute RIII, but at the critical stage, phylum Proteobacteria, genera *Escherichia-Shigella* and *Eubacterium xylanophilum_group*, and species *Lactobacillus murinus* were identified as potential biomarkers. The dysbiosis in gut microbiota was persistent until one week after radiation, showing a decrease in the diversity of gut microbiota and in the relative abundance of beneficial bacteria, such as *Lactobacillus*, and an increase in the relative abundance of opportunistic pathogens. The supplementation of probiotics could alleviate the acute RIII in mice, and partially restored the diversity of gut microbiota and its dysbiosis. This provides new insights to identify potential bacteria taxa as diagnostic biomarkers and new targets for the intervention against RIII.

## Materials and Methods

### Animals and Radiation Protocol

The animal model was established as previously described (Li et al., 2019). Male 6-8 weeks-old C57BL/6J mice (SLAC Laboratory Animal Co. Ltd, Shanghai, China) were maintained under specific pathogen-free conditions with free access to drinking water and diet and standard conditions (ambient temperature 22 ± 2°C, air humidity 30-60% and a 12/12-h light/dark cycle). The animal study was reviewed and approved by the Animal Ethics Committee of Soochow University. Radiation were given *via* a ^60^Co γ source at a dose rate of 2 Gy/min. For studies, investigating the effects of radiation on gut microbiota, the mice were randomly assigned into 4 groups and treated with TBI, which was delivered by a ^60^Co γ source with the single doses of 0, 4, 8, and 12 Gy. For probiotics intervention study, the mice were randomly assigned to 4 groups: non-radiated control group, probiotics treatment group, 9 Gy TBI group and probiotics treatment + 9 Gy TBI group. Each group contained 8-10 mice for survival experiments and 5-6 mice per time point for sample collection experiments.

### Schedule for the Intervention of Compound Probiotics

Compound probiotics, containing Lactobacillus acidophilus LA85, Lactobacillus rhamnosus LRa05, Lactobacillus plantarum Lp90, Bifidobacterium longum BL21 and Bifidobacterium lactis BLa80, were treated with three-layer embedding technology of anti-gastric acid, anti-oxidation and anti-physical extrusion and supplied by Jiangsu Wecare biotechnology company (Suzhou, China), which were stored at -20°C in the form of freeze-dried powder. 0.1 g of powder, containing 10 billion colony forming units, was dissolved in phosphate-buffered saline, and was orally gavage-administered to each mouse for 10 days before 9 Gy TBI and then 4 days post TBI. Maltodextrin was served as control and orally administered to each mouse in the control groups as the same regimen.

### Histo-Pathological Study

The intestinal tissue samples were collected at the indicated times after TBI. For histological assessment, the tissue samples were fixed in 10% neutral formalin, embedded in paraffin, cut into 3-μm thick sections, and stained with hematoxylin and eosin (H&E) or periodic acid-Schiff (PAS) as previously described (Li et al., 2019). All the slides were observed under a light microscope (Olympus Corp., Tokyo, Japan) and images were taken. For the measurement of villus height and crypt depth, no less than 30 well-oriented intact crypt-villus units were measured for each mouse using Image J 1.43 software. For studying their immunohistochemistry (IHC), the paraffin sections were de-paraffinized, rehydrated, incubated with 3% hydrogen peroxide for 15 min to quench the endogenous peroxidase and then boiled in sodium citrate buffer (pH 6.0) for antigen retrieval. After non-specific antigen blocking with 5% bovine serum albumin for 30 min at 37°C, the sections were incubated overnight with primary antibody against Ki67 (1:400, Cell Signaling Technology, Beverly, MA, USA) and then secondary antibody (Zhongshan Golden Bridge Biotechnology, Beijing, China) for 1 h at 37°C. For observation, the sections were treated with 3, 3N-diaminobenzidine tertrahydrochloride for color development and hematoxylin for counterstaining.

### TdT Mediated dUTP Nick End Labeling (TUNEL) Assay

The TUNEL assay was used to localize apoptotic cells in the crypts. The paraffin tissue sections, collected at 6 h after TBI, were treated according to the instructions of *in situ* Cell Death Detection Kit (Roche Diagnostic, Mannheim, Germany) as reported previously (Li et al., 2019). The apoptotic cells, exhibiting green fluorescence, were counted in no less than 30 well-oriented crypts per mouse.

### *16S rRNA* Gene Sequencing

The fecal samples of each mouse were freshly collected at indicated times after TBI, and then immediately put into sterile plastic tubes on ice and frozen at -80℃ until *16S rRNA* gene sequencing analysis. After total genomic DNA extraction using sodium dodecyl sulfate-based method, the V3-V4 hyper-variable regions of *16S rRNA* gene were amplified using universal primers (343F: 5′-TACGGRAGGCAGCAG-3′; 798R: 5′-AGGGTATCTAATCCT-3′), and then purified with Agencourt AMPure XP beads (Beckman Coulter Co., Brea, CA, USA), which were finally sequenced on an Illumina Miseq sequencing platform (Illumina Inc., San Diego, CA, USA) with two paired-end read cycles of 300 bases each. The raw paired-end reads were trimmed using Trimmomatic software and the de-noised, reads with 75% of bases above Q20 were retained using QIIME software (version 1.8.0). The further chimeric sequences were removed using VSEARCH to generate clean reads from which the operational taxonomic units were clustered at 97% identity. The representative reads, selected by QIIME package, were annotated by BLAST against Silva database (Version 123) using RDP classifier (confidence threshold was set to 70%). The sequencing of *16S rRNA* gene amplicons were conducted by OE Biotech Co., Ltd. (Shanghai, China). The raw data were uploaded to National Center for Biotechnology Information Sequence Read Archive database under the BioProject accession numbers PRJNA668669 and PRJNA699974.

### Sequencing Data Analysis

The analysis of alpha and beta diversities were performed using QIIME. The microbial diversity in each group was estimated using alpha diversity, including Chao1 and Shannon indices. The microbial diversity among different groups was estimated using beta diversity analysis *via* Principal coordinates analysis (PCoA) with phylogeny-based (UniFrac) unweighted distances and hierarchical clustering analysis with unweighted pair group method with arithmetic mean (UPGMA). The differences in relative abundances at different taxonomic levels were identified using Kruskal Wallis test and Linear discriminant analysis (LDA) effect size (LEfSe).

### Statistical Analysis

GraphPad Prism v7.0 (GraphPad Software, Inc., San Diego, CA, USA) was used for the analyses and graph preparation. For graph data, the results were expressed as mean ± standard error. Survival analysis was performed using Kaplan-Meier survival testing. The statistical comparisons between two groups were analyzed using unpaired two-tailed Student’s *t*-tests. Linear correlations were calculated using Pearson’s correlation coefficient. The associations within the abundances of microbial taxa was computed using Spearman’s correlation distance and visualized in heat map. The significance threshold of *P* < 0.05, unless otherwise stated, was applied in all the cases of multiple hypothesis testing by Benjamini-Hochberg false discovery rate (FDR) correction.

## Results

### Establishment of Mouse Models for Acute RIII

**Figure 1:**
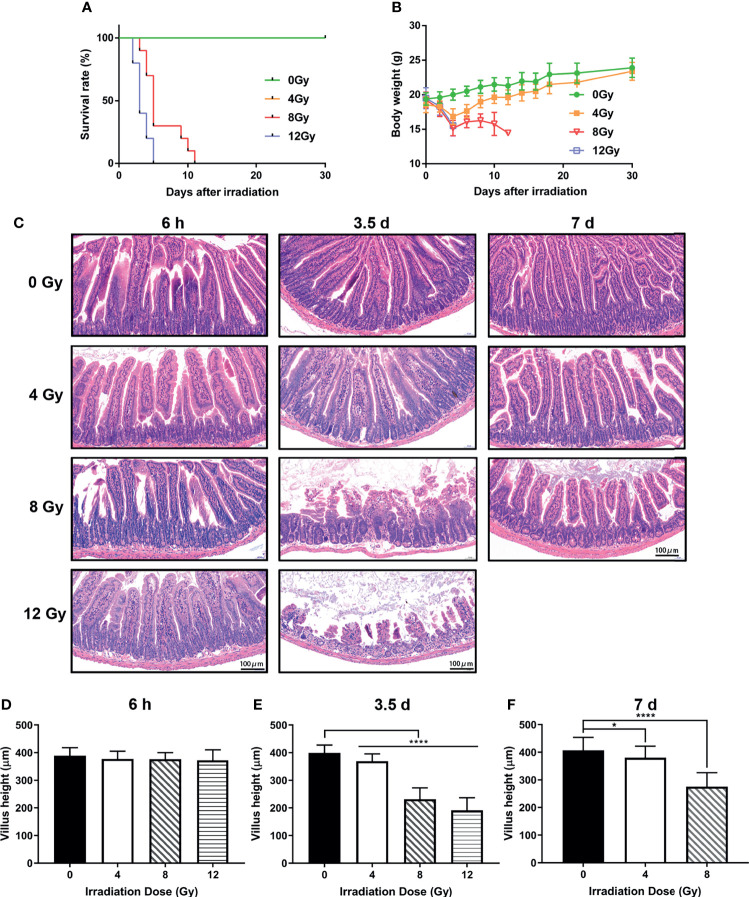
Effects of different doses of radiation on survival and intestinal injuries of C57 mice. **(A)** Kaplan-Meier survival analysis of mice after 0, 4, 8 and 12 Gy TBI. **(B)** Body weights of radiated mice in each group after 0, 4, 8 and 12 Gy TBI. **(C)** Representative H&E stained images of intestinal tissues harvested at 6 h, 3.5 d and 7 d following radiation. **(D–F)** Bar graph of the villus height determined from panel **(C)** (n = 6/group; * indicates *P <* 0.05; **** indicates *P <* 0.001).

**Figure 2:**
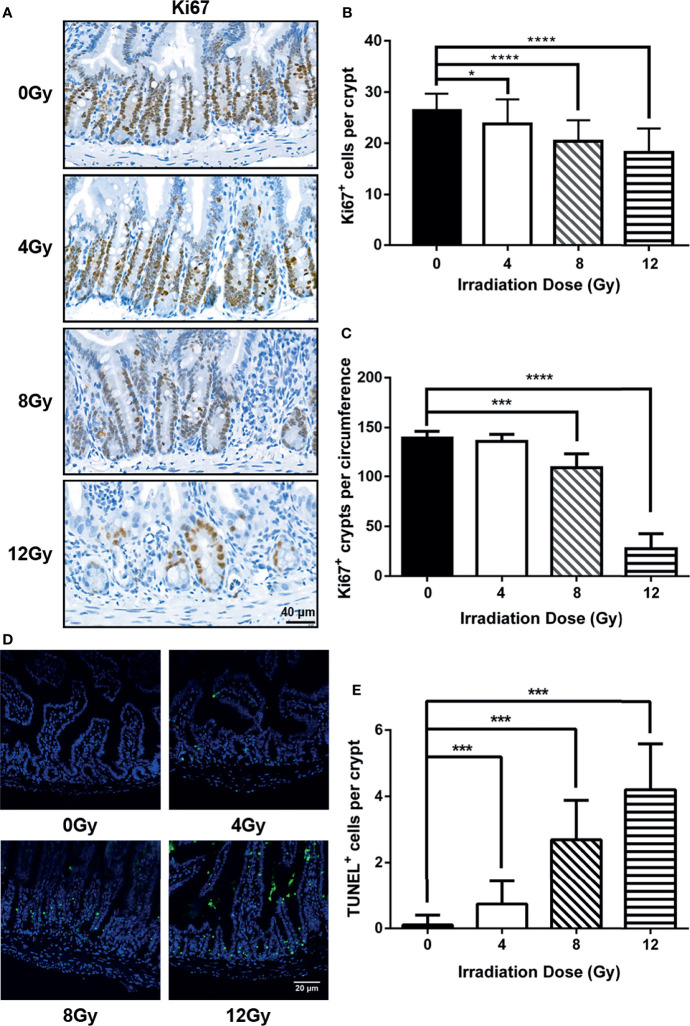
Injury degree of small intestine was dependent with radiation doses in mouse models. **(A)** The representative IHC images for the expression of Ki67 in small intestine from radiated mice were compared with that from the control mice at 3.5 d post radiation. The number of **(B)** Ki67 positive cells in each crypt and **(C)** Ki67 positive crypts per circumference determined from Ki67-immunostained sections of small intestines. At least 30 well-oriented crypts per mouse were counted. The crypt, having more than 10 Ki67 positive cells, was identified as a Ki67 positive crypt. **(D)** Representative images from intestine at 6 h post radiation were shown for apoptotic cells determined by TUNEL assay. **(E)** Number of apoptotic cells in each crypt determined from TUNEL stained sections of small intestines. (n = 6/group; * indicates *P <* 0.05; *** indicates *P <* 0.001; **** indicates *P <* 0.0001).

In order to comprehensively study the effects of IR on gut microbiota, the mouse models were established using TBI for 0, 4, 8 and 12 Gy. First, the survival condition and weight loss were monitored for 30 days after radiation. At day 3.5 post radiation, the weight of mice, which received 4 Gy TBI, reached to the lowest value, and recovered to normal similar to that of non-radiated mice at day 30 and all survived, while the mice, which received 8 Gy and 12 Gy TBI could not recover their weight to normal and died within 13 and 5 days, respectively (**Figures 1A, B**). Then, the intestinal tissues were harvested at 6 h, 3.5 d and 7 d post radiation. The histo-pathological examination illustrated that there was no obvious injury in crypt-villi architecture as visualized by H&E at 6 h post-radiation (different doses) (**Figures 1C, D**), while an increased number of apoptotic cells in crypt were observed in TUNEL assay (**Figures 2D, E**). At day 3.5 post radiation, a noticeable shortening in the villus height in H&E staining (**Figures 1C, E**) and a decrease in the number of Ki67 positive proliferating cells in each crypt and Ki67 positive crypts per circumference in IHC analysis were observed (**Figures 2A–C**). These parameters at 3.5 d post radiation were dependent on radiation doses. At day 7 post-radiation, the injury of epithelium in surviving mice was restored rapidly after the doses of 4 and 8 Gy radiation, manifesting an increase in the villus height (**Figures 1C, F**). In brief, in this study, the radiation doses of 4, 8 and 12 Gy caused different degrees of intestinal injuries, and the time points of 6 h, 3.5 d and 7 d post radiation represented the prodromal stage, critical stage and recovery stage of acute RIII, respectively.

### Alterations in the Diversity and Composition of Gut Microbiota Post-Radiation

**Figure 3:**
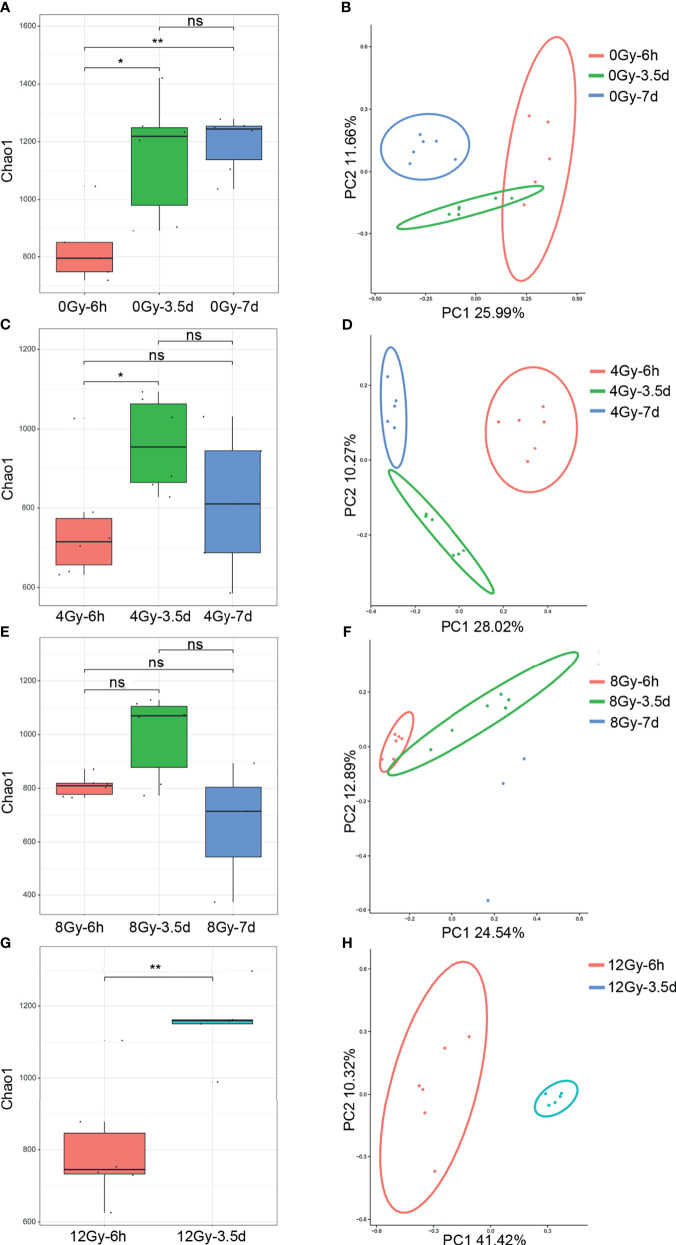
Diversity of gut microbiota of mice following 0, 4, 8 and 12 Gy TBI changed with the fecal collection time in Chao1 index and PCoA analysis. The **(A)** Chao1 and **(B)** PCoA of microbial communities from the control mice at 6 h, 3.5 d and 7 d. The **(C, E, G)** for Chao1 indices and **(D, F, H)** for the PCoA of gut microbiota from 4, 8 and 12 Gy radiated mice at 6 h, 3.5 d and 7 d (n = 6/group; * indicates *P <* 0.05; ** indicates *P <* 0.01); ns indicates no significance.

**Figure 4:**
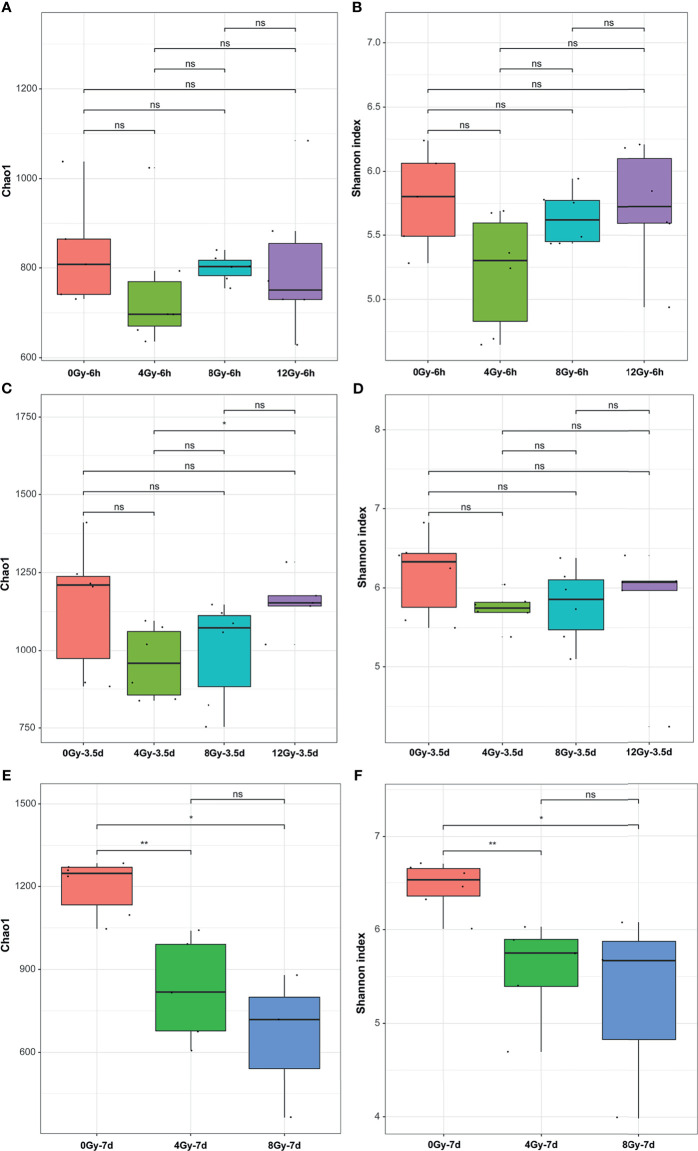
Effects of different doses of radiation on the bacterial diversity in alpha diversity analysis are shown (within-sample diversity). **(A, C, E)** Chao1 indices and **(B, D, F)** Shannon indices of the microbial alpha diversity from control and radiated C57 mice at 6 h, 3.5 d and 7 d after exposure to TBI doses of 4, 8 and 12 Gy. (n = 6/group; * indicates *P <* 0.05; ** indicates *P <* 0.01); ns indicates no significance.

**Figure 5:**
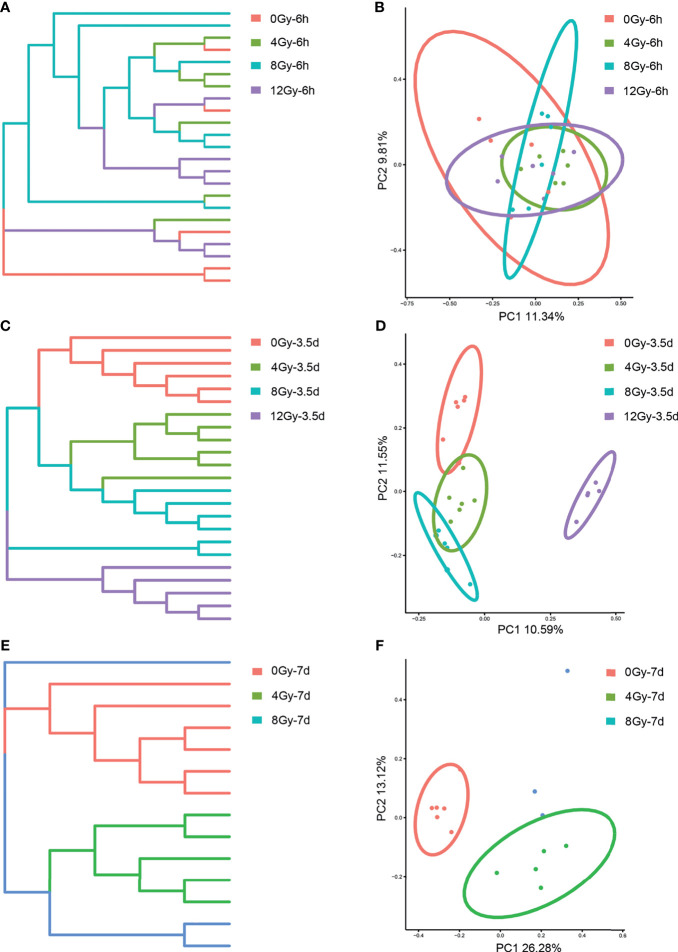
Effects of different doses of radiation on gut microbiota in beta diversity analysis are shown (between-sample diversity). **(A, C, E)** UPGMA tree and **(B, D, F)** PCoA of the gut microbiota from control and radiated C57 mice at 6 h, 3.5 d and 7 d after exposure to TBI doses of 4, 8 and 12 Gy.

The *16S rRNA* gene sequencing technology was used to detect the alterations in the diversity and composition of gut microbiota associated with acute RIII in fecal samples harvested at 6 h, 3.5 d and 7 d, following 0, 4, 8 and 12 Gy radiation in mouse models. The richness and evenness of gut microbiota and complexity of its structure were evaluated by Chao1 and Shannon indices in the alpha diversity analysis. The PCoA and UPGMA analyses are commonly used in the beta diversity analysis to study the repeatability of samples within a group and similarities and differences among different groups. Considering the diversity of gut microbiota changed with the biological rhythm, control groups were set up for each sample collection time. It was observed that, for control mice, the structure of gut microbiota in fecal samples obtained at different time points was completely different (**Figures 3A, B**), while that for different time points post the same radiation dose showed the similar alteration (**Figures 3C–H**). We conjectured that the alteration was influenced by biological rhythm. The fecal samples collected at the same time point were analyzed thereafter. Both the alpha and beta diversity analyses showed no significant differences in the diversities of gut microbiota among different radiation dose groups at 6 h (**Figures 4A, B** and **5A, B**). At 3.5 d post-radiation, although no significant differences were observed among groups by alpha diversity analysis (**Figures 4C, D**), the PCoA plots and UPGMA analysis revealed clear differences (**Figures 5C, D**), indicating the correlation of gut microbiota with radiation doses. At 7 d post radiation, both the Chao1 and Shannon indices of radiation groups showed a significant decrease as compared to the control group (**Figures 4E, F**). The beta diversity analysis revealed distinguished features of gut microbiota between the radiation and control groups, without obvious correlation with radiation dose (**Figures 5E, F**). In short, the gut microbiota responded to the different doses of radiation at day 3.5 post radiation and did not recover at day 7 post radiation.

**Figure 6:**
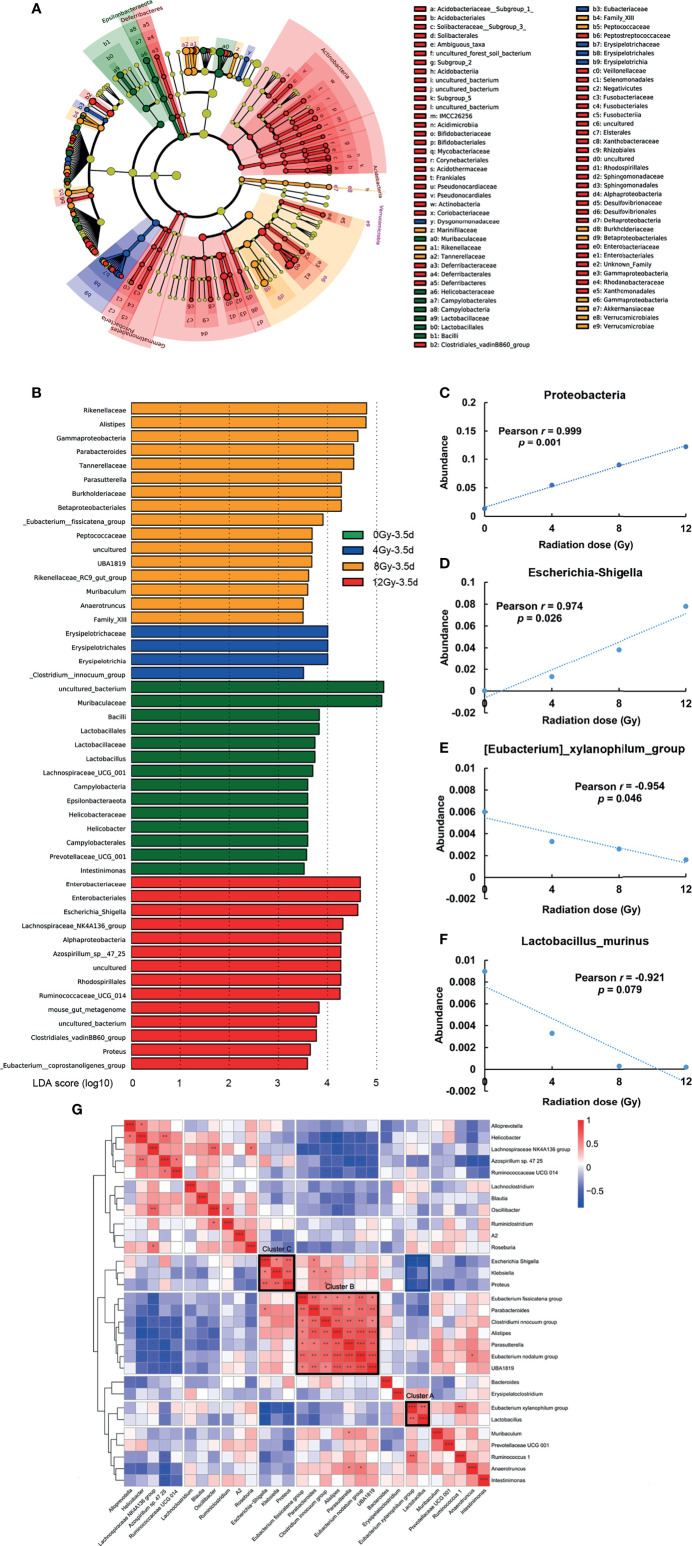
Bacterial markers associated with radiation doses at 3.5 d post radiation. **(A, B)** LEfSe analysis of key taxa, showing differences in the composition of gut microbiota among mice radiated with different doses. The characteristic taxonomies having LDA scores higher than 3.5 were selected and presented. Pearson correlation revealed that the relative abundance of **(C)** *Proteobacteria*, **(D)** *Escherichia-Shigella*, **(E)** *Eubacterium xylanophilum_group*, and **(F)** *Lactobacillus murinus* had the linear correlation with different radiation doses at 3.5 d post radiation. **(G)** Top 30 most abundant genera at 3.5 d post radiation were clustered by Spearman’s correlation distance. Three poly-microbial clusters with high covariance (intra cluster mean Spearman’s ρ > 0.5) and LDA scores of higher than 3.5 are presented in a heat map. (n = 6/group; * indicates *P <* 0.05; ** indicates *P <* 0.01; *** indicates *P <* 0.001).

The LEfSe analysis was used to further explore the characteristics of gut microbiota at day 3.5 post different radiation doses, which revealed a heterogeneous array of abundance profiles at different taxonomic levels, following different radiation doses. The characteristic taxonomies with LDA score higher than 3.5 are presented in **Figures 6A, B**. The phylum Epsilonbacteraeota and families *Muribaculaceae*, *Lactobacillaceae*, and *Helicobacteraceae* showed the highest relative abundances in the gut microbiota of control mice. The mice radiated with 8 Gy showed the classes Gammaproteobacteria and Verrucomicrobiae, and families *Rikenellaceae*, *Burkholderiaceae*, *Tannerellaceae* and *Akkermansiaceae* to be the highest relative abundant taxa of this group. The phylum Actinobacteria and family *Enterobacteriaceae* were the dominated taxa in the mice group radiated with 12 Gy. The results at genus level were more informative (**Table 1**). The genera *Lactobacillus*, *Lachnospiraceae UCG 001*, *Helicobacter* and *Prevotellaceae UCG 001* showed the highest relative abundances in the gut microbiota of control mice, while the highest relative abundances in that of mice groups radiated with 4, 8 and 12 Gy were as following: *Clostridium innocuum* in 4 Gy; *Alistipes*, *Parasutterella*, *Parabacteroides*, *Eubacterium fissicatena group* in 8 Gy; and *Escherichia Shigella*, *Lachnospiraceae NKRA136 group*, *Azospirillum.sp 47 25*, *Ruminococcaceae UCG014*, and *Proteus* in 12 Gy.

**Table 1:** The summary from phylum to genus of bacteria taxa with LDA scores of higher than 3.5 and their clusters.

Group	Cluster	Phylum	Class	Order	Family	Genus	LDA score	P value
0Gy-3.5d		Bacteroidetes	Bacteroidia	Bacteroidales	Muribaculaceae	Muribaculum	5.14	0.00181
0Gy-3.5d	A	Firmicutes	Bacilli	Lactobacillales	Lactobacillaceae	Lactobacillus	3.74	0.00093
0Gy-3.5d		Firmicutes	Clostridia	Clostridiales	Lachnospiraceae	Lachnospiraceae UCG 001	3.70	0.00149
0Gy-3.5d		Epsilonbacteraeota	Campylobacteria	Campylobacterales	Helicobacteraceae	Helicobacter	3.60	0.00152
0Gy-3.5d		Bacteroidetes	Bacteroidia	Bacteroidales	Prevotellaceae	Prevotellaceae UCG 001	3.57	0.00688
0Gy-3.5d		Firmicutes	Clostridia	Clostridiales	Ruminococcaceae	Intestinimonas	3.52	0.01536
4Gy-3.5d	B	Firmicutes	Erysipelotrichia	Erysipelotrichales	Erysipelotrichaceae	Clostridium innocuum group	3.51	0.00095
8Gy-3.5d	B	Bacteroidetes	Bacteroidia	Bacteroidales	Rikenellaceae	Alistipes	4.78	0.00103
8Gy-3.5d	B	Bacteroidetes	Bacteroidia	Bacteroidales	Tannerellaceae	Parabacteroides	4.53	0.00108
8Gy-3.5d	B	Proteobacteria	Gammaproteobacteria	Betaproteobacteriales	Burkholderiaceae	Parasutterella	4.28	0.00085
8Gy-3.5d	B	Firmicutes	Clostridia	Clostridiales	Lachnospiraceae	Eubacterium fissicatena group	3.91	0.00776
8Gy-3.5d	B	Firmicutes	Clostridia	Clostridiales	Ruminococcaceae	UBA1819	3.68	0.00235
8Gy-3.5d		Bacteroidetes	Bacteroidia	Bacteroidales	Rikenellaceae	Rikenellaceae RC9 gut group	3.61	0.04234
12Gy-3.5d	C	Proteobacteria	Gammaproteobacteria	Enterobacteriales	Enterobacteriaceae	Escherichia Shigella	4.61	0.01565
12Gy-3.5d		Firmicutes	Clostridia	Clostridiales	Lachnospiraceae	Lachnospiraceae NKRA136 group	4.31	0.03840
12Gy-3.5d		Proteobacteria	Alphaproteobacteria.	Rhodospirillales	uncultured	Azospirillum.sp 47 25	4.27	0.00008
12Gy-3.5d		Firmicutes	Clostridia	Clostridiales	Ruminococcaceae	Ruminococcaceae UCG014	4.26	0.04558
12Gy-3.5d		Bacteroidetes	Bacteroidia	Bacteroidales	Muribaculaceae	mouse gut metagenome	3.83	0.00025
12Gy-3.5d	C	Proteobacteria	Gammaproteobacteria	Enterobacteriales	Enterobacteriaceae	Proteus	3.65	0.00807
12Gy-3.5d		Firmicutes	Clostridia	Clostridiales	Ruminococcaceae	Eubacterium coprostanoligenes group	3.59	0.00348

On the other hand, the Pearson correlation analysis was used to identify bacteria, having linear correlation with radiation dose. In particular, the relative abundances of phylum Proteobacteria (**Figure 6C**), genera *Escherichia-Shigella* (**Figure 6D**) and *Eubacterium xylanophilum_group* (**Figure 6E**), and species *Lactobacillus murinus* (**Figure 6F**) showed linear correlation (*P* < 0.05, except for *Lactobacillus murinus P* < 0.1) with radiation dose at day 3.5, and showed the value of Pearson Correlation Coefficient > 0.9, making them promising biomarkers for the evaluation of the degree of acute RIII.

The interactions among different microbes, such as symbiosis and competition, are the key elements in the maintenance of healthy gut microbiota, which can be affected by gut environment and *vice versa* (Pickard et al., 2017). We questioned whether there were unknown interplays among bacteria existed in this model. Accordingly, the top 30 most abundant genera in all the samples were clustered at day 3.5 post radiation using Spearman’s correlation distance and presented in the form of a heat map (**Figure 6G**). Interestingly, the three poly-microbial Clusters A, B and C (intra cluster mean Spearman’s ρ > 0.5, FDR < 0.05) were formed by genera with strong positive correlations. The Clusters A, B and C were mainly composed of the characteristic bacteria of the control group, 4 and 8 Gy radiation groups, and 12 Gy radiation group in LEfSe analysis, respectively (**Table 1**). According to the literature, the Clusters A, B and C showed the predominance of beneficial bacteria, opportunistic pathogens, and pathogenic bacteria, respectively (Wallen et al., 2020). Furthermore, there were significant negative correlations among several genera from these clusters, such as *Lactobacillus* in Cluster A and *Escherichia-Shigella* in Cluster C (ρ = -0.76, FDR = 0.0001), and *Lactobacillus* in Cluster A and *Proteus* in Cluster C (ρ = -0.55, FDR = 0.03).

In summary, these results suggested that the acute RIII was accompanied by the dysbiosis of gut microbiota, including a decrease in its diversity and relative abundance of beneficial bacteria, and an increase in the relative abundance of pathogens. Furthermore, the dysbiosis was persistent until the recovery stage of acute RIII, which indicated that the interventions might be needed to recover this dysbiosis.

### Compound Probiotics Attenuates the Intestinal Epithelium Injury and Partially Restores the Gut Microbiota of Radiated Mice

**Figure 7:**
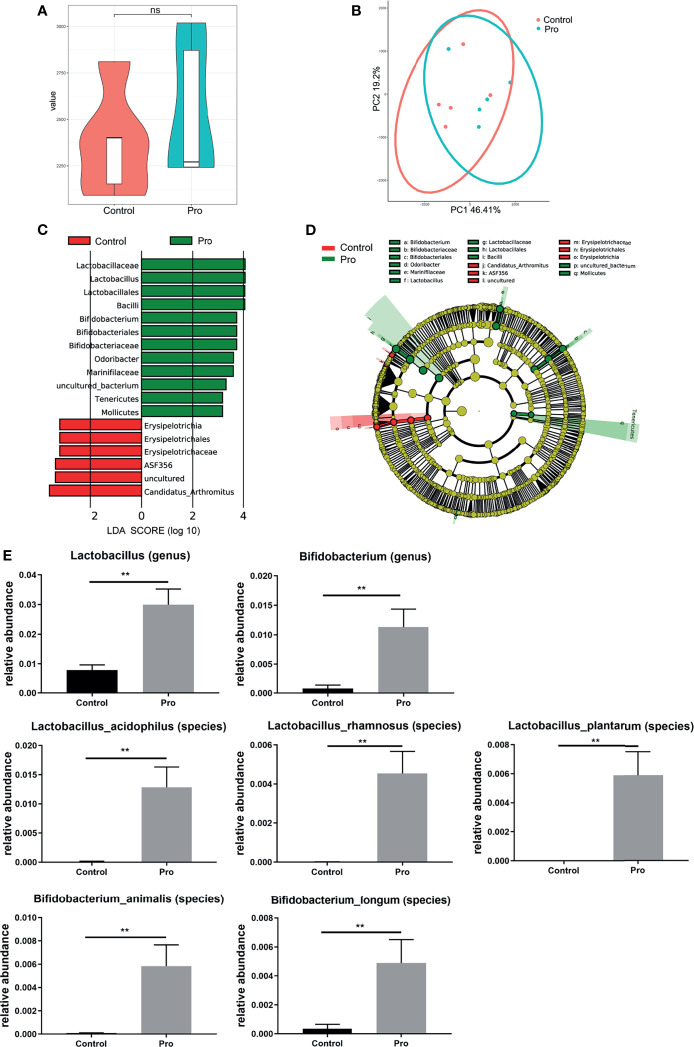
Patterns of gut microbiota after pretreatment with probiotics for 14 days in mouse model. The richness and composition of gut microbiota were analyzed by **(A)** Chao1 index and **(B)** PCoA. **(C, D)** Differentially abundant bacteria between Control (red) and probiotics pretreatment group (green) are presented in a histogram of LDA scores using LEfSe. **(E)** The relative abundance of differentially abundant bacteria taxa in gut microbiota between groups after pretreatment with probiotics. (n = 5/group; ** indicates *P <* 0.01); ns indicates no significance.

**Figure 8:**
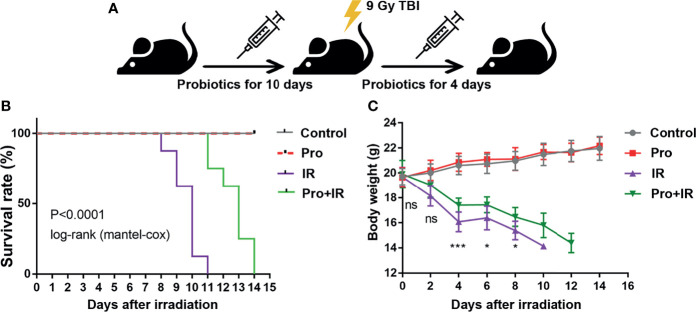
Compound probiotics increased the survival time and reduced the weight loss of radiated mice. **(A)** Experimental schedule for the induction of gut injuries by radiation and intervention with probiotics. C57 BL/6J mice received oral gavage of PBS or probiotics for 10 consecutive days before 9 Gy TBI and 4 consecutive days after TBI. **(B)** Kaplan-Meier survival analysis of mice after 9 Gy TBI. **(C)** Body weights of radiated mice in each group after 9 Gy TBI (n = 8/group; *indicates *P <* 0.05; *** indicates *P <* 0.001).

**Figure 9:**
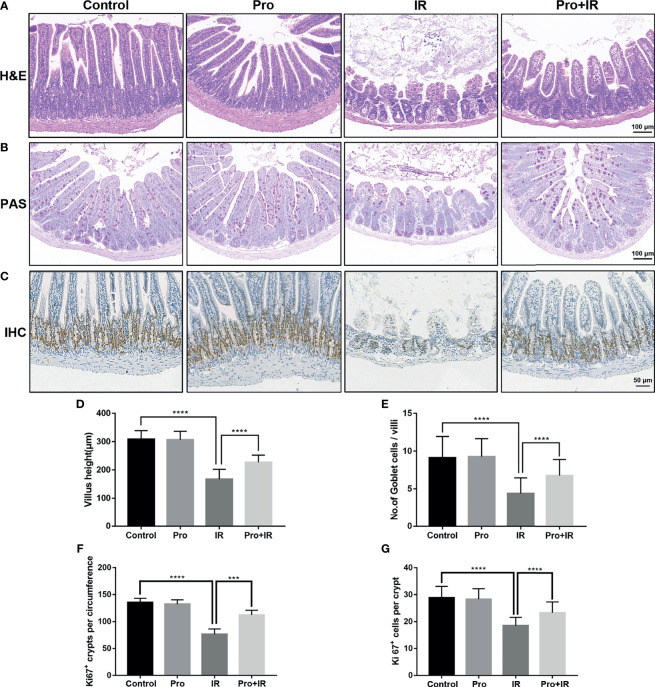
Compound probiotics alleviated the intestinal epithelium injury in RIII. Intestinal tissues were harvested at 4 d following radiation. Representative images from histological staining by **(A)** H&E, **(B)** PAS and **(C)** IHC for Ki67. Histogram showing the **(D)** villus height, **(E)** number of Goblet cells per villi, **(F)** numbers of Ki67 positive crypts per circumference and **(G)** Ki67 positive cells in each crypt. (n = 5/group; *** indicates *P <* 0.001; **** indicates *P <* 0.0001).

The attenuation of acute RIII using the supplement compound probiotics, including *Lactobacillus acidophilus*, *Lactobacillus rhamnosus*, *Lactobacillus plantarum*, *Bifidobacterium longum* and *Bifidobacterium lactis* was investigated. After the administration of compound probiotics for 14 days in mouse model, although the richness and composition of gut microbiota were not obviously affected (**Figures 7A, B**), the relative abundances of genera *Lactobacillus* and *Bifidobacterium* were found to be significantly increased (**Figures 7C–E**), suggesting that the exogenous probiotics could survive passage through the gastrointestinal tract. After the continuous intra-gastric administration for 10 days before 9 Gy TBI and 4 days after TBI (**Figure 8A**), the probiotics increased the survival time of mice (**Figure 8B**) and reduced their weight loss (**Figure 8C**). As compared to the radiation group, the probiotics improved the shortening of villus height (**Figures 9A, D**) and increased the number of goblet cells (**Figures 9B, E**), as well as the numbers of surviving crypts across the intestinal section and Ki67 positive cells in each crypt (**Figures 9C, F, G**) post radiation. Therefore, these results showed that the probiotics could protect intestinal epithelium and improve the proliferation of intestinal crypt cells in radiated mice.

**Figure 10:**
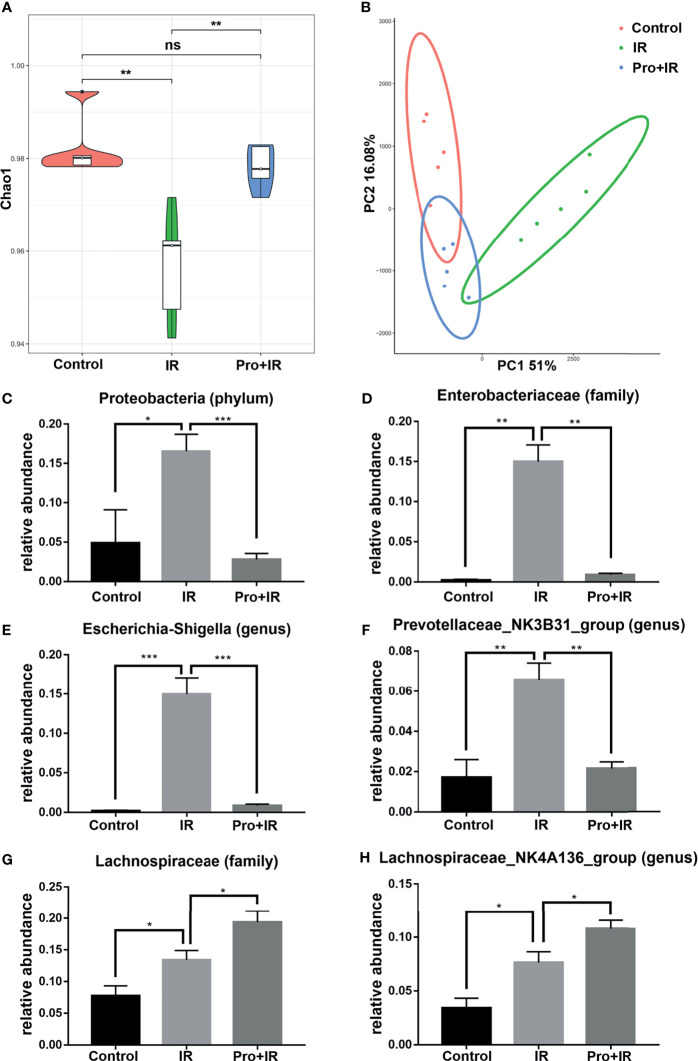
Compound probiotics restored the diversity and composition of gut microbiota in radiated mice. The fecal samples were harvested at 4 d following IR. **(A)** Chao1 index in alpha diversity analysis and **(B)** PCoA in beta diversity analysis of gut microbiota in each group are shown. The relative abundance of **(C)** *Proteobacteria*, **(D)** *Enterobacteriaceae*, **(E)** *Escherichia-Shigella*, **(F)** *Prevotellaceae NK3B31*, **(G)** *Lachinospiraceae* and **(H)** *Lachinospiraceae NK4A136* in each groups. (n = 5/group; * indicates *P <* 0.05; ** indicates *P <* 0.01; *** indicates *P <* 0.001); ns indicates no significance.

In order to explore the effects of probiotics on the gut microbiota of radiated mice, the fecal samples from each group were collected and analyzed using the *16S rRNA* gene sequencing. The Chao1 index showed that the probiotics restored the richness and structural complexity of the gut microbiota of radiated mice (**Figure 10A**). The PCoA analysis showed differences in the gut microbiota among groups (**Figure 10B**). As compared to the radiation group, the probiotics reduced the relative abundances of phylum Proteobacteria, family *Enterobacteriaceae*, genera *Escherichia-Shigella* and *Prevotellaceae NK3B31*, and increased that of family *Lachinospiraceae* and genus *Lachinospiraceae NK4A136* (**Figures 10C–H**). Therefore, these results suggested that the probiotics could partially restore the diversity and composition of gut microbiota in radiated mice.

## Discussion

At present, there is no globally-accepted definition of what a ‘healthy gut microbiota’ is. The term dysbiosis is described as an ill-defined state of gut microbiota, typically correlated with the loss of diversity of microbiota and specific beneficial symbionts and bloom of potentially harmful pathobionts (Sommer et al., 2017; Mullish et al., 2021). The pathobionts usually interact with their host symbiotically, but can become pathogenic under certain conditions (Mullish et al., 2021). The alterations in the composition of gut microbiota can offer resistance or assistance to infections by pathogens in gut (Bäumler and Sperandio, 2016). Although facing a continuous perturbation, such as the changes in dietary patterns or even a catastrophic one, the gut microbiota remains relatively stable and restores its functional state in a healthy host, owing to a marked capacity for self-regeneration, termed as resilience (Sommer et al., 2017). The diversity of gut microbiota is a key requirement for its resilience. In other words, the complex and diverse microbiota, containing many species, is less susceptible to perturbation. The reason is as the limited resources are being competed by different species, the well-adapted species limit the influx or overgrowth of other species, including pathobionts (Bäumler and Sperandio, 2016; Pickard et al., 2017). On contrary, a decrease in the diversity of gut microbiota (dysbiosis) might cause many intestinal diseases (Ott et al., 2004; Manichanh et al., 2006; Chang et al., 2008; Willing et al., 2010; Lepage et al., 2011; Sokol et al., 2017; Mentella et al., 2020). However, in most of the cases, the intervention by modulating the microbiota is still in the exploratory stage. This could be due to the fact that the feature of dysbiosis differs for each disease, and are influenced differently by the severity and development of a certain disease (Mullish et al., 2021), urging the need for the development of individually-tailored intervention strategies. Therefore, unravelling the feature of dysbiosis in certain diseases will result in the better understanding of pathogenesis and development of precise strategies for the manipulation of gut microbiota against certain diseases.

In this study, the mouse models were used to delineate the signatures of gut microbiota in the progression of acute RIII after exposure to the different doses of 4, 8 and 12 Gy radiation. Using the *16S rRNA* gene sequencing technology, the diversity of gut microbiota was found to be not significantly shifted at the prodromal stage of acute RIII, while the overall composition of gut microbiota was dependent on the radiation dose at the critical stage of acute RIII. The LEfSe analysis showed differences in the relative abundance of bacterial phylotypes among different doses of radiation. Among them, a decrease in the relative abundance of beneficial *Lactobacillus* and an increase in that of pathogenic *Escherichia Shigella* were shown to have linear correlation with radiation doses, while there was a negative correlation between them as showed by Spearman’s association analysis. At the recovery stage of acute RIII, the diversity of gut microbiota decreased and the dysbiosis was persistent. The intervention by compound probiotics protected the intestinal epithelium, promoted the proliferation of intestinal crypt cells, and partially restored the diversity and composition of gut microbiota in radiated mice.

The results of this study align with previous studies and broaden the understanding of the effects of radiation on gut microbiota. As shown in this study, the dysbiosis caused by radiation has been reported to be associated with reduction in alpha diversity and a common increase in the diversity of phylum Proteobacteria (Gerassy-Vainberg et al., 2018). At the genus level, reduction in the relative abundances of *Lactobacillus* and *Prevotella*, and an increase in that of *Alistipes* and *Parabacteroides* post radiation were shown, which were consistent with the previous studies (Kim et al., 2015; Cui et al., 2017; Gerassy-Vainberg et al., 2018). Besides, a similar time-specific pattern of gut microbiota was found in the control and radiation groups, which might be explained by circadian rhythmicity, a determinant factor to shape the composition of gut microbiota (Thaiss et al., 2014). Therefore, in the absence of accurate illustration about the time point of sample collection, the comparison of the abundances of taxa among different studies becomes controversial.

Although there has been considerable efforts focusing on the identification of biomarkers for studying the effects of high-dose IR, yet accessible biomarkers are still needed to be identified for assessing the extent and predicting the outcome of injury in acute RIII. As an accessible sample, feces, providing information on both the status of the intestinal tract and gut microbiota, are supposed to provide potential biomarkers for the early diagnosis of RIII (Reis Ferreira et al., 2019). However, in this study, the diversity of gut microbiota was not significantly shifted at 6 h post different doses of radiation. The radiation dose-dependent changes in the relative abundance of specific taxa appeared at 3.5 d after IR. Therefore, the gut microbiota was considered to be potential biomarker at the critical stage of acute RIII. Under normal circumstances, the microbes in gut persistently colonize intestine and perform symbiotic functions (Lynch and Pedersen, 2016; Oliphant and Allen-Vercoe, 2019; Dupont et al., 2020). The maintenance of beneficial gut microbiota requires a homeostatic balance between the microbiota and gut barrier (Sommer et al., 2017). Once the gut barrier is breached by radiation, it affects the intestinal environment as well as the composition of gut microbiota. The dose-dependent bacterial patterns might be explained by the dose-dependent intestinal injuries.

Gerassy-Vainberg S *et al.* found that germ-free mice transplanted with postradiation microbiota were predisposed to radiation injury (Gerassy-Vainberg et al., 2018), indicating that there might be some harmful factors in postradiation microbiota. In our study, an increase in the relative abundance of distinctive opportunistic pathogens or pathogens were observed in the critical stage of acute RIII. There is limited literature available on their gut pathophysiological functions as commensal to human gastrointestinal tracts, but if the relative abundance of these opportunistic pathogens increases, they might be harmful to their host, especially causing intestinal inflammation or infections (Falony et al., 2016; Moschen et al., 2016; Chia et al., 2017; Chen et al., 2018; Chia et al., 2018; Dong et al., 2018; Kim et al., 2018; Koh et al., 2018; Lopetuso et al., 2018; Aroca-Ferri et al., 2019; Ju et al., 2019; Wang K. et al., 2019). In inflammatory bowel disease (IBD), it was reported that the relative abundance of pathobiont *Escherichia coli* increased in gut microbiota, which might play a role in the pathogenesis of IBD (Mirsepasi-Lauridsen et al., 2019). The genus *Parasutterella* is involved in the maintenance of bile acid and cholesterol metabolism (Ju et al., 2019). However, the increase in the relative abundance of *Parasutterella* was considered to be correlated with irritable bowel syndrome (Chen et al., 2018). The genus *Alistipes* may have protective role against colitis and liver fibrosis (Parker et al., 2020). In contrast, an increase in the abundance of *Alistipes* is suggested to be pathogenic in ileitis (Moschen et al., 2016; Parker et al., 2020) and colorectal cancer (Moschen et al., 2016). *Parabacteroides distasonis* is defined as a member of 18 core gut microbiota in humans (Falony et al., 2016), having anti-inflammatory and metabolic benefits (Koh et al., 2018; Wang K. et al., 2019), while its elevated levels could contribute to IBD (Lopetuso et al., 2018). *Clostridium innocuum* has been reported to be associated with peritonitis (Aroca-Ferri et al., 2019), antibiotic associated diarrhea and severe colitis (Chia et al., 2018) and extra-intestinal clostridial infection (Chia et al., 2017). All the genera in Cluster C are the pathogens from family *Enterobacteriaceae*. *Escherichia-Shigella* can invade colonic epithelium and provoke strong inflammatory responses, leading to the destruction of colonic epithelium, which then results in bacillary dysentery (Belotserkovsky and Sansonetti, 2018). The *Proteus* species have low-abundance in the commensal human gut microbiota, which have pathogenic potential in IBD and urinary tract infections (Hamilton et al., 2018). *Klebsiella pneumoniae* is a well-known pathogen, causing several human infections (Prokesch et al., 2016; Kaur et al., 2018; Wyres et al., 2020). It was worthy to be noted in this study that the *Klebsiella* genus had strong positive correlation with pathogens elevated after 12 Gy radiation, indicating a higher risk for antimicrobial-resistant infections. In this study, the relative abundance of these pathogens increased as compared to that of control group, but its potential roles in the pathogenesis of acute RIII still needs to be evaluated.

In our study, it was observed that the beneficial genera were reduced and showed negative correlations with the harmful genera. The *Lactobacillus* species colonize almost all sites in the gastrointestinal tract and is a well-known probiotic, known for its multiple benefits, such as promoting the regeneration of intestinal epithelial cells through the production of lactic acid (Lee et al., 2018). *E. xylanophilum* plays essential roles in the fermentation of insoluble fibers in human large intestine (Duncan et al., 2016). The genus *Muribaculum* was more abundant in non-radiated mice as compared to the radiated ones, and is known for its versatility of the transformation of bile acids (Marion et al., 2020) and degradation of complex carbohydrates (Lagkouvardos et al., 2019). The interplay within microbial communities affects the gut microbiota (Sommer et al., 2017). The negative correlation between *Lactobacillus* and *Escherichia Shigella* in this study suggested that the reduction in the relative abundance of beneficial bacteria facilitate the proliferation of pathogens.

Recent studies have focused on the restoration of beneficial bacteria *via* the therapeutic supplementation of probiotics to alleviate RIII (Lee et al., 2018; Riehl et al., 2019). The probiotics, containing *Lactobacillus* spp., has been proved to benefit mice by accelerating the regeneration of intestinal stem cells after RIII (Lee et al., 2018; Riehl et al., 2019) and has also been proved to benefit patients to reduce the incidence of radiotherapy-induced diarrhea (Devaraj et al., 2019). However, not all the studies have shown positive results (Giralt et al., 2008). In our study, the protective effects of probiotics were confirmed in acute RIII, which included increasing the number of goblet cells and protecting the proliferation of crypt cells. Goblet cells can secrete intestinal mucus layers, which act as the frontline host defense against irritants and microbial attachment and invasion (Kim and Ho, 2010). Besides, this study found that the probiotics could partially restore the diversity and composition of gut microbiota in radiated mice, which included a decrease in the relative abundance of harmful *Escherichia-Shigella* and an increase in that of radio-protective *Lachinospiraceae* (Guo et al., 2020). A study reported that the beneficial bacteria regulated the composition of gut microbiota by competing for nutrients or producing metabolites, and affected the colonization of intestinal pathogens (Bron et al., 2017; Pickard et al., 2017). For example, *Lactobacillus reuteri* can produce metabolites, having broad-spectrum antibacterial activity, which directly inhibit the growth of intestinal pathogens (Cleusix et al., 2007). Therefore, it opens a door for the invention strategies of beneficial bacteria to limit the expansion of pathogen in acute RIII.

A limitation of this study is that the findings are based on observation only, showing the association, and not providing explanation for their mechanism. In order to move beyond the correlations, the discovery of causal microbes in the pathogenesis of RIII requires experimental validation *via* microbe-phenotype triangulation approach (Surana and Kasper, 2017) in future studies. Another limitation is the limited knowledge of bacterial function involved currently. For instance, *Ruminococcaceae* and *Lachnospiraceae* are the two predominant butyrate-producing families (Bach Knudsen et al., 2018). However, the abundances of varying genera in butyrate-producing families were elevated after 4, 8 and 12 Gy radiation, which indicated the existence of extra specific functions in acute RIII at genus level. No doubt, the identification of bacterial functions *via* experimental studies will enable us to better understand the functional shifts in acute RIII.

In conclusion, the gut bacteria present opportunities to evaluate and prevent the pathogenesis of acute RIII. We report that the acute RIII is accompanied by dysbiosis of gut microbiota, including their decreased diversity, a reduction in the relative abundance of beneficial bacteria and an increase in that of pathogens. The gut microbiota cannot be used as sensitive biomarkers at the prodromal stage of acute RIII, but are suggested to be potential biomarkers in the critical stage of acute RIII. The dysbiosis is persistent until the recovery stage of acute RIII, and interventions may be needed to restore the gut microbiota to normal. Probiotics affects the development of acute RIII as well as intestinal bacterial profiles. This study provides new insights to identify potential bacterial taxa as diagnostic biomarkers in acute RIII and a conceptual basis for the exploration in the contribution of pathogens to the pathogenesis of RIII and using microbiota-targeting therapeutic approaches to alleviate RIII.

## Data Availability Statement

The datasets presented in this study can be found in online repositories. The names of the repository/repositories and accession number(s) can be found in the article/supplementary material.

## Ethics Statement

The animal study was reviewed and approved by the Animal Ethics Committee of Soochow University.

## Author Contributions

ML and YT conceived and designed the study. T-SZ, L-WX, and SC coordinated and performed most experimental work. SF embedded the probiotics. L-FT and J-YX provided the histological analysis. HZ and CY performed statistical analyses. ML and T-SZ wrote the manuscript, and YT provided critical review. All authors contributed to the article and approved the submitted version.

## Funding

This work was supported by Jiangsu Provincial Key Project in Research and Development of Advanced Clinical Technique (BL2018657), Jiangsu Medical Innovation Team (CXDT-37), Medicine Outstanding Leader of Suzhou (62), Pre-research Program of the 2th Affiliated Hospital of Soochow University (SDFEYBS1701), Scientific Research Program for Young Talents of China National Nuclear Corporation (51003), XKTJ-RC202007, Military Medical Science and Technology Youth Training Program (17QNP029).

## Conflict of Interest

Author SF was employed by company Wecare Probiotics (Suzhou) Co., Ltd.

The remaining authors declare that the research was conducted in the absence of any commercial or financial relationships that could be construed as a potential conflict of interest.

## Publisher’s Note

All claims expressed in this article are solely those of the authors and do not necessarily represent those of their affiliated organizations, or those of the publisher, the editors and the reviewers. Any product that may be evaluated in this article, or claim that may be made by its manufacturer, is not guaranteed or endorsed by the publisher.

## Abbreviations

ARS, acute radiation syndrome; FDR, false discovery rate; H&E, hematoxylin and eosin; IBD, inflammatory bowel disease; IHC, immunohistochemistry; IR, ionizing radiation; LDA, Linear discriminant analysis; LEfSe, LDA effect size; PAS, periodic acid-Schiff; PCoA, Principal coordinates analysis; RIII, radiation-induced intestinal injury; TBI, total body irradiation; TUNEL, TdT mediated dUTP Nick End Labeling; UPGMA, unweighted pair group method with arithmetic mean.
